# Molecular network analysis of human microRNA targetome: from cancers to Alzheimer’s disease

**DOI:** 10.1186/1756-0381-5-17

**Published:** 2012-10-03

**Authors:** Jun-ichi Satoh

**Affiliations:** 1 Department of Bioinformatics and Molecular Neuropathology, Meiji Pharmaceutical University, 2-522-1 Noshio, Kiyose, Tokyo, 204-8588, Japan

**Keywords:** Alzheimer’s disease, Cancer, MicroRNA, Molecular network, Targetome

## Abstract

MicroRNAs (miRNAs), a class of endogenous small noncoding RNAs, mediate posttranscriptional regulation of protein-coding genes by binding chiefly to the 3’ untranslated region of target mRNAs, leading to translational inhibition, mRNA destabilization or degradation. A single miRNA concurrently downregulates hundreds of target mRNAs designated “targetome”, and thereby fine-tunes gene expression involved in diverse cellular functions, such as development, differentiation, proliferation, apoptosis and metabolism. Recently, we characterized the molecular network of the whole human miRNA targetome by using bioinformatics tools for analyzing molecular interactions on the comprehensive knowledgebase. We found that the miRNA targetome regulated by an individual miRNA generally constitutes the biological network of functionally-associated molecules in human cells, closely linked to pathological events involved in cancers and neurodegenerative diseases. We also identified a collaborative regulation of gene expression by transcription factors and miRNAs in cancer-associated miRNA targetome networks. This review focuses on the workflow of molecular network analysis of miRNA targetome *in silico*. We applied the workflow to two representative datasets, composed of miRNA expression profiling of adult T cell leukemia (ATL) and Alzheimer’s disease (AD), retrieved from Gene Expression Omnibus (GEO) repository. The results supported the view that miRNAs act as a central regulator of both oncogenesis and neurodegeneration.

## Introduction

MicroRNAs (miRNAs) constitute a class of endogenous small noncoding RNAs conserved through the evolution. They mediate posttranscriptional regulation of protein-coding genes by binding chiefly to the 3^′^ untranslated region (3^′^UTR) and occasionally to the 5^′^UTR or coding regions of target mRNAs [1]. This interaction leads to translational inhibition, mRNA destabilization or degradation, depending on the degree of sequence complementarity. During miRNA biogenesis, the pri-miRNAs are transcribed from the intra- and inter-genetic regions of the genome by RNA polymerase II, followed by processing by the RNase III enzyme Drosha into pre-miRNAs. After nuclear export, they are cleaved by the RNase III enzyme Dicer into mature miRNAs that consist of approximately 22 nucleotides. Finally, a single-stranded mature miRNA is selectively recruited onto the Argonaute-containing RNA-induced silencing complex (RISC), where the seed sequence located at positions 2 to 8 from the 5^′^ end of the miRNA serves as an essential scaffold for recognizing the target mRNA [2]. Furthermore, recent evidence indicates that Argonaute proteins directly regulate miRNA processing by binding to pri-miRNA transcripts in the nucleus [3].

Currently, 1,600 precursor and 2,042 mature human miRNAs are registered in miRBase Release 19 (August 2012). In general, a single mRNA is targeted by several different miRNAs, while a single miRNA at one time reduces the production of hundreds of target proteins that constitute “targetome” [4]. Thus, redundant interactions between miRNAs and their targets result in the complexity of miRNA-regulated gene expression. Furthermore, certain miRNAs activate transcription and translation of the targets, further enhancing the complexity [5,6]. Consequently, the whole human “microRNAome (miRNAome)” regulates greater than 60% of all protein-coding genes [7]. By targeting multiple transcripts and affecting expression of numerous proteins, miRNAs are capable of fine-tuning diverse cellular functions, including development, differentiation, proliferation, apoptosis and metabolism [2]. Therefore, aberrant regulation of miRNA expression is greatly involved in pathological events underlying cancers [8] and neurodegenerative diseases, such as Alzheimer’s disease (AD) and Parkinson’s disease (PD) [9,10]. Certain miRNAs are released extracellularly, and circulate steadily in the serum, plasma, and cerebrospinal fluids, which clinically serve as diagnostic and prognostic disease biomarkers [11].

Recent advances in systems biology have made a great breakthrough by illustrating the cell-wide map of complex molecular interactions with the aid of the literature-based knowledgebase of molecular pathways [12]. The logically arranged molecular networks construct the whole system characterized by robustness, which maintains the proper function of the system in the face of genetic and environmental perturbations [13]. Actually, miRNAs play an active role in conferring robustness to various biological systems by reinforcing the transcriptional machinery to reduce random fluctuations in gene expression [14]. In the scale-free molecular network, targeted disruption of limited numbers of critical components designated hubs, on which the biologically important molecular interactions concentrate, efficiently disturbs the whole cellular function by destabilizing the network [15]. Importantly, an individual miRNA often targets the hub gene in the human protein-protein interaction (PPI) network [16]. Therefore, the identification and characterization of hub molecules in the miRNA targetome network would help us to elucidate biological roles of individual miRNAs.

Until recently, the question remains unanswered whether the miRNA targetome regulated by an individual miRNA generally constitutes the biological network of functionally-associated molecules or simply reflects a random set of functionally-independent genes. To address this issue, we attempted to characterize the molecular network of the whole human miRNA targetome [17]. We found that the set of highly reliable targets for approximately 20% of all human miRNAs constructed biologically meaningful molecular networks, supporting the view that the miRNA targetome generally constitutes the biological network of functionally-associated molecules in human cells. Notably, we identified a collaborative regulation of gene expression by transcription factors and miRNAs in cancer-associated miRNA targetome networks, indicating that the human miRNAome plays a specialized role in regulation of oncogenesis [17]. More recently, we have characterized the molecular network of experimentally validated targets for hundreds of miRNAs whose expression is downregulated in AD brains [18]. We found that aberrant cell cycle progression owing to deregulation of miRNA targetome networks plays a central role in the pathogenesis of AD.

The present review focuses on the workflow of an *in silico* approach how to effectively identify biological roles of individual miRNAs through molecular network analysis of the miRNA targetome. Here, we would show its application to representative datasets of cancers and AD.

## Workflow of molecular network analysis of MicroRNA targetome

### Preparation of MicroRNA dataset

**Figure 1:**
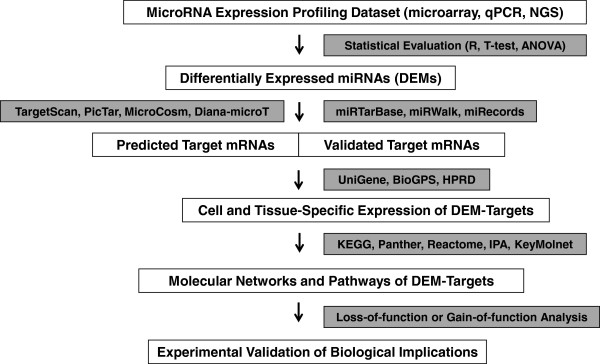
** The workflow of molecular network analysis of microRNA targetome.** First, differentially expressed miRNAs (DEMs) among distinct samples and experimental conditions are extracted from microRNA expression profiling datasets based on microarray, qPCR, and next-generation sequencing (NGS) experiments by the standard statistical evaluation. Next, predicted targets and/or validated targets for DEMs are obtained by using target prediction programs, such as TargetScan, PicTar, MicroCosm and Diana-microT 3.0, or by searching them on databases of experimentally validated targets, such as miRTarBase, miRWalk, and miRecords. The expression of DEM targets in the cells and tissues examined is verified by searching them on UniGene, BioGPS, and HPRD. Molecular networks and pathways relevant to DEM targets are identified by using pathway analysis tools, such as KEGG, IPA, and KeyMolnet. Finally, the functionally inverse relationship between miRNAome and targetome is validated by loss-of-function or gain-of-function experiments in an *in vitro* and/or *in vivo* model.

First of all, we prepare the list of miRNAs whose function we attempt to characterize (Figure 1). For the whole human miRNAome, we could retrieve the complete list from miRBase Release 19 ( http://www.mirbase.org), as described previously [17]. For the selection of focused miRNAome, we could download microRNA expression profiling datasets from Gene Expression Omnibus (GEO) repository ( http://www.ncbi.nlm.nih.gov/geo). They are derived from experimental data performed on microarray, quantitative RT-PCR (qPCR), and high-throughput sequencing. In the next step, we extract a set of differentially expressed miRNAs (DEMs), either upregulated or downregulated among distinct samples and/or different experimental conditions, following statistical evaluation with Bioconductor on R statistical package ( http://www.r-project.org), and so on. 

### MicroRNA target prediction

In general, miRNAs could form an energetically stable Watson-Crick base pair with target mRNAs [2]. In most occasions, the seed sequence located at positions 2 to 8 from the 5^′^ end of the miRNA serves as an essential scaffold for recognizing the target mRNA in the condition of a perfect seed match with miRNA recognition element (MRE) sequences of mRNA. Target sites often avoid the sequences immediately after the stop codon, which have the possibility of falling into the ribosome shadow [19]. The thermodynamic rule and the evolutional conservation of MRE sequences make it possible to fairly accurately predict miRNA target mRNAs by computational approaches [2]. Open source miRNA target prediction programs, including TargetScan version 6.2 ( http://www.targetscan.org), PicTar (pictar.mdc-berlin.de), MicroCosm version 5 ( http://www.ebi.ac.uk/enright-srv/microcosm), miRanda ( http://www.microrna.org), and Diana-microT version 3.0 (diana.cslab.ece.ntua.gr/microT), are mostly armed with unique algorithms that survey MRE sequences in the 3^′^UTR of target mRNAs. As a result, the predicted targets vary greatly among the distinct programs utilized [20]. Increasing evidence suggests that MRE sequences are located occasionally in the 5^′^UTR or coding sequences (CDS) [21,22], both of which are ignored by the conventional prediction programs. Furthermore, predicted targets are usually cell- and tissue-type non-specific. These drawbacks confer a substantial risk for detecting numerous false positive and negative ones. The integration of the results from several prediction programs, along with examination of tissue-specific interactions, might provide an advantage for reducing unreliable targets to some extent [23,24].

Recently, several databases of experimentally validated miRNA targets are established to overcome the unreliability of target prediction (Figure 1). The miRecords database (mirecords.biolead.org) includes 2,286 records of experimentally validated interactions between 548 miRNAs and 1,579 target genes derived from 9 species extracted after thorough literature curation, accompanied with the storage of predicted targets collected from datasets of 11 established miRNA target prediction programs [25]. The miRTarBase (mirtarbase.mbc.nctu.edu.tw) represents the collection of 4,270 manually curated miRNA-target interactions validated experimentally between 669 miRNAs and 2,533 target genes among 14 species [26]. It is followed by a clear description of experimental methods for target validation on each interaction, such as luciferase reporter assay, western blot, quantitative RT-PCR, and microarray experiments. The miRWalk database ( http://www.umm.uni-heidelberg.de/apps/zmf/mirwalk) contains both predicted and validated information on miRNA-target interactions focused on 449 human biological pathways and 2,356 disorders of Online Mendelian Inheritance in Man (OMIM) [27]. Predicted targets are originated based on its own algorithm that covers MRE sequences located both inside and outside the 3^′^UTR of target mRNAs, and are also collected from datasets of 8 established miRNA target prediction programs. Validated targets are identified by an automated text-mining search on PubMed to extract experimentally validated miRNA-target interactions, including those involved in miRNA processing, followed by PubMed article identifiers (PMID).

### In silico validation of tissue-specific expression of MicroRNA target mRNAs

Although experimentally validated targets represent a source of reliable candidates, it is worthless when they are not expressed in the cells and tissues examined. Most simply, we could verify the expression of target mRNAs in specified tissues and cells by analyzing them on UniGene ( http://www.ncbi.nlm.nih.gov/unigene), an organized view of the transcriptome that evaluates semi-quantitatively the expression sequence tag (EST) calculated as the number of transcripts per million (TPM) (Figure 1) [18]. We could investigate mRNA expression levels based on microarray data in specified tissues and cells by searching them on a gene annotation resource named BioGPS (biogps.org) [28]. Similarly, H-Invitational Database (H-InvDB) ( http://www.h-invitational.jp) includes the Human Anatomic Gene Expression Library (H-ANGEL) that provides gene expression data from microarray experiments and EST profiles determined on a panel of normal adult human tissues [29]. Human Protein Reference Database (HPRD) ( http://www.hprd.org) linked to NCBI Entrez is also useful to identify the tissue-specific expression of proteins and their subcellular location.

### Genome-wide analysis of MicroRNA target mRNAs

The simultaneous assessment of miRNA and mRNA expression profiles provides a rational approach to identify a set of miRNAs whose expression levels are negatively correlated with the levels of their target mRNAs [30-32]. However, it is often difficult to determine the optimum experimental time required for miRNA-induced degradation of target mRNAs, because time lags exist in expression changes between miRNAs and target mRNAs. Time course-dependent profiles of miRNA-mRNA expression make it possible to more exactly identify the inverse relationship between relevant miRNAs and mRNAs [33]. However, the interaction of a miRNA with a target mRNA does not always cause mRNA degradation. Instead, it often leads to reduction in protein expression levels by translational repression.

Recently, the methods of quantitative proteomics are established to overcome the difficulties attributable to the dissociation of miRNA and mRNA dynamics. They include stable isotope labeling with amino acids in culture (SILAC), isobaric tag for relative and absolute quantitation (iTRAQ), and two-dimensional difference gel electrophoresis (2D-DIGE), all of which are combined with miRNA expression profiling [34-36]. Nevertheless, these techniques could not exclude indirect alterations of protein expression. To enrich a class of mRNAs directly bound to the RISC complex, the method designated as ribonucleoprotein immunoprecipitation (IP) followed by GeneChip (RIP-Chip) has been established. By this advanced technique, a previous study has characterized miRNA target mRNAs recovered from the Ago2-IP fraction of Hodgkin lymphoma cells [37]. They found that approximately 40% of miRNA target transcripts are derived from targets for abundantly co-expressed miRNAs in the cells, although this technique could not specify the exact pair of miRNAs and their target mRNAs.

A recent progress in the next-generation sequencing (NGS) technology has revolutionized the field of genomic research. Currently, we could efficiently identify endogenous miRNAs and target mRNAs on a genome-wide scale at one time by using the method named as high-throughput sequencing of RNAs isolated by crosslinking immunoprecipitation (HITS-CLIP-Seq) or alternatively by the improved version designated as the photoactivatable-ribonucleoside-enhanced crosslinking immunoprecipitation (PAR-CLIP-Seq) [38,39]. In both of them, the RISC complex components comprising miRNAs, mRNAs, and RISC proteins are crosslinked by ultraviolet (UV) prior to immunoprecipitation with an antibody specific for the RISC component protein. Then, deep sequencing data are processed for target prediction programs to identify interaction sites between miRNAs and target mRNAs. By these techniques, a previous study showed that MRE sequences are located in 3^′^UTR (40%), 5^′^UTR (1%), CDS (25%), intron (12%), intergenic regions (6%), and non-coding RNA (4%), respectively, in the postnatal mouse neocortex [38]. A different study revealed that the GCACUU motif, enriched in 3^′^UTR and CDS of target mRNAs, matches the seed of a miRNA family that constitutes 68% of entire miRNAs in mouse embryonic stem cells (mESCs) [40].

### Molecular network analysis of MicroRNA target mRNAs

To identify biologically relevant molecular networks and pathways extracted from high-throughput data, we could analyze them by using a battery of bioinformatics tools for analyzing molecular interactions on the comprehensive knowledgebase (Figure 1). They include Kyoto Encyclopedia of Genes and Genomes (KEGG) ( http://www.kegg.jp), Panther ( http://www.pantherdb.org), Reactome ( http://www.reactome.org), Ingenuity Pathways Analysis (IPA) (Ingenuity Systems, http://www.ingenuity.com), and KeyMolnet (Institute of Medicinal Molecular Design, http://www.immd.co.jp). KEGG, Panther, and Reactome are open sources, whereas IPA and KeyMolnet are commercial ones, all of which are updated frequently. After July 1, 2011, the KEGG FTP site for academic users has been transferred from GenomeNet at Kyoto University to NPO Bioinformatics Japan. Therefore, the FTP access is currently available only to paid subscribers, although the publicly funded domain is freely accessible at GenomeNet. This review focuses on the application of KEGG, IPA, and KeyMolnet to molecular network analysis.

KEGG includes manually curated reference pathways that cover a wide range of metabolic, genetic, environmental, and cellular processes, and human diseases [41]. Currently, KEGG contains 198,560 pathways generated from 428 reference pathways. When importing of Entrez Gene IDs into the Functional Annotation tool of Database for Annotation, Visualization and Integrated Discovery (DAVID) version 6.7 (david.abcc.ncifcrf.gov), DAVID identifies the most relevant KEGG pathway and gene ontology (GO) categories, composed of the genes enriched in the given set, followed by an output of statistical significance evaluated by the modified Fisher’s exact test [42].

IPA is a knowledgebase that contains approximately 3,000,000 biological and chemical interactions and functional annotations with definite scientific evidence, curated by expert biologists. By uploading the list of Gene IDs and expression values into the Core Analysis tool, the network-generation algorithm identifies focused genes integrated in a global molecular network. IPA calculates the score p-value that reflects the statistical significance of association between the genes and the networks by the Fisher’s exact test.

KeyMolnet contains knowledge-based contents on 150,500 relationships among human genes and proteins, small molecules, diseases, pathways and drugs, curated by expert biologists [12,17]. They are categorized into the core contents collected from selected review articles and textbooks with the highest reliability or the secondary contents extracted from PubMed abstracts and Human Reference Protein database (HPRD). By importing the list of Gene IDs and expression values, KeyMolnet automatically provides corresponding molecules as a node on networks. The neighboring network-search algorithm selects one or more molecules as starting points to generate the network of all kinds of molecular interactions around starting molecules, including direct activation/inactivation, transcriptional activation/repression, and the complex formation within the designated number of paths from starting points. The generated network is compared side by side with 484 human canonical pathways, 892 diseases, and 219 pathological events of the KeyMolnet library (the April 2012 version). The algorithm counting the number of overlapping molecular relations between the extracted network and the canonical pathway makes it possible to identify the canonical pathway showing the most significant contribution to the extracted network [12,17].

### Experimental validation of biological implications

Molecular network analysis enables us to characterize the most relevant networks and pathways involved in the miRNA targetome *in silico*. When the expression of DEMs is downregulated, theoretically, the targetome is predicted to be upregulated, and presumably hyperactivated under pathological conditions. In contrast, when the expression of DEMs is upregulated, the targetome is predicted to be downregulated, and possibly hypoactivated under disease conditions. The functionally inverse relationship between miRNAs in the miRNAome and mRNAs in the targetome should be validated by loss-of-function or gain-of-function experiments by introducing antagomirs (anti-sense miRNAs) or premir oligonucleotides in an *in vitro* and/or *in vivo* model in an adequate setting (Figure 1). This step is highly important but often labor intensive. For example, a recent study by using microarray and qPCR showed that the expression of a set of miRNAs, most robustly miR-206, are upregulated in Tg2576 AD transgenic mice and human AD brain samples [43]. Importantly, intraventricular injection of a miR-206 antagomir restored decreased levels of brain-derived neurotrophic factor (BDNF), a highly likely target of miR-206, followed by a remarkable improvement of memory function.

## Molecular network of MicroRNA targetome

### Human MicroRNAome plays a specialized role in oncogenesis

Recently, we studied the molecular network of the whole human miRNA targetome [17]. The complete set of human miRNAs was downloaded from miRBase Release 16. Among 1,223 human miRNAs examined, Diana-microT 3.0 predicted the targets from 532 miRNAs (43.5%). This program calculates the miRNA-targeted gene (miTG) score that reflects the weighted sum of the scores of all conserved and non-conserved MRE sequences on the 3^′^UTR of the target mRNA [44]. To optimize the parameter of miRNA-target interaction, we considered target genes with a cutoff of the miTG score ≥ 20 as highly reliable targets. Among 532 miRNAs, we identified 273 miRNAs with highly reliable targets. Among 273 miRNAs, KeyMolnet successfully extracted targetome networks from 232 miRNAs that consist of 19% of the whole human miRNAome. Thus, these results supported the view that the human miRNA targetome regulated by an individual miRNA generally constitutes the biological network of functionally-associated molecules [17]. Therefore, it is possible that even small changes in the expression of a single miRNA could affect a wide range of signaling pathways and networks involved in diverse biological functions.

**Figure 2:**
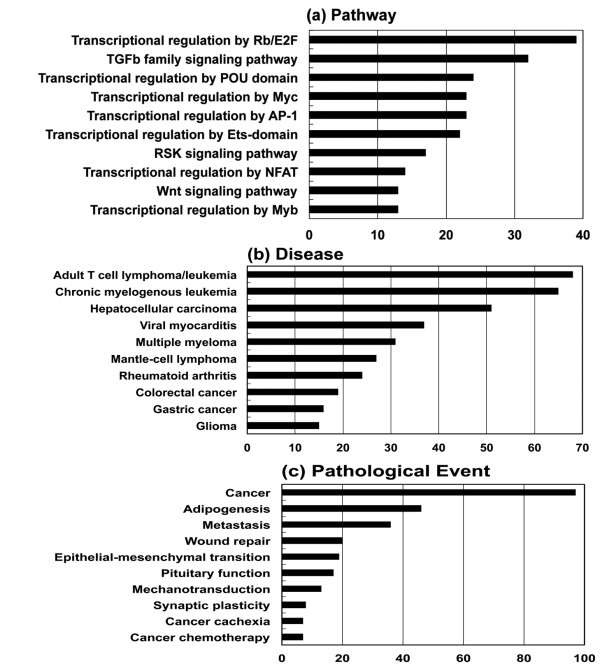
** The whole human microRNAome plays a specialized role in oncogenesis.** Among 1,223 miRNAs of the whole human miRNAome, Diana-microT 3.0 identified the set of reliable targets from 273 miRNAs. Among them, KeyMolnet extracted molecular networks from 232 miRNAs. The generated network was compared side by side with human canonical networks of the KeyMolnet library, composed of 430 pathways, 885 diseases, and 208 pathological events to identify the canonical network showing the most statistically significant contribution to the extracted network. After top three pathways, diseases, and pathological events were individually totalized, the cumulated numbers of top 10 of (**a**) pathway, (**b**) disease, and (**c**) pathological event categories are expressed as a bar graph. The figure is cited from our study [17].

Next, the generated network was compared side by side with human canonical networks of the KeyMolnet library, composed of 430 pathways, 885 diseases, and 208 pathological events (the July 2010 version). When top three pathways, diseases, and pathological events were individually totalized, the most relevant pathway was ‘transcriptional regulation by retinoblastoma (Rb) protein/transcription factor E2F’ (n = 39; 6.8% of total), followed by ‘TGF-beta (TGFb) family signaling pathway’ (n = 32; 5.6%) and ‘transcriptional regulation by POU domain factor’ (n = 24; 4.2%), the most relevant disease was ‘adult T cell lymphoma/leukemia’ (n = 68; 12.1%), followed by ‘chronic myelogenous leukemia’ (n = 65; 11.5%) and ‘hepatocellular carcinoma’ (n = 51; 9.1%), and the most relevant pathological event was ‘cancer’ (n = 97; 24.7%), followed by ‘adipogenesis’ (n = 46; 11.7%) and ‘metastasis’ (n = 36; 9.2%) (Figure 2) [17]. Thus, we found that the human miRNAome plays a specialized role in regulation of oncogenesis. 

The protooncogene c-myb is a key transcription factor for development of normal hematopoietic cells and neoplasms. Recent evidence indicates that miR-15a targets c-myb, while c-myb binds to the promoter of miR-15a, providing an autoregulatory feedback loop in human hematopoietic cells [45]. Consistent with these observations, we found ‘transcriptional regulation by myb’ as the most relevant pathway to the miR-15a targetome network [17]. These results propose a scenario that miR-15a synchronously downregulates both c-myb itself and downstream genes transcriptionally regulated by c-myb, resulting in more effective inactivation of the whole miR-15a targetome network governed by the hub gene c-myb.

The Rb/E2F pathway acts as a gatekeeper for G1/S transition in the cell cycle. The Rb/E2F-regulated G1 checkpoint control is frequently disrupted in cancer cells. A previous study showed that miR-106b directly regulates E2F1 at a posttranscriptional level [46]. E2F1 activates transcription of miR-106b, while miR-106b targets E2F1, constituting a negative feedback loop in gastric cancer cells. Consistent with these observations, we identified ‘transcriptional regulation by Rb/E2F’ as the most relevant pathway to the miR-106b targetome network [17]. Again, it is possible that miR-106b simultaneously downregulates both E2F family transcription factors and downstream genes transcriptionally regulated by E2F, resulting in efficient inactivation of the whole miR-106b targetome network governed by the hub molecule E2F. Thus, there exists a complex crosstalk between miRNAs and E2F family proteins, and it plays a crucial role in regulation of oncogenic signaling [47].

These results suggest an existence of collaborative regulation of gene expression by transcription factors and miRNAs in cancer-associated miRNA targetome networks. This concept is supported by a recent study showing that the collaborative regulation of gene expression involves a feedforward loop of coordinated regulation by miRNAs and transcription factors [48]. The crosstalk between miRNAs and transcription factors in the human protein interaction network is categorized into four regulatory modules, comprising single-regulation, co-regulation, crosstalk, and independent [49]. Furthermore, co-expressed miRNAs often share transcription factors and function in a cooperative manner to regulate common biological processes [50].

To protect the cells from oncogenic insults, the transcription factor p53 acts as “the guardian of the genome” by regulating a battery of target genes involved in cell cycle arrest, apoptosis, senescence, and DNA repair. Therefore, deregulation of tumor suppressor function of p53 is closely associated with oncogenesis. We found ‘transcriptional regulation by p53’ as the most relevant pathway to the target network of all let-7 family members except for let-7d [17]. p53 regulates the expression of a panel of miRNAs and the components of the miRNA-processing machinery, such as Drosha, DGCR8, Dicer, and TARBP2, all of which have p53-reponsive elements in their promoters [51,52]. Furthermore, Dicer and TARBP2, along with p53, serve as a target for the let-7 family miRNAs, suggesting a pivotal interplay between p53 and let-7 in miRNA biogenesis. The expression of let-7 family members was greatly reduced in a panel of cancer cells [53].

Zinc finger transcription factors ZEB1 and ZEB2 act as a transcriptional repressor of E-cadherin. The expression of miR-200b, which targets both ZEB1 and ZEB2, was downregulated in the cells that undergo TGFb-induced epithelial-mesenchymal transition (EMT), and was lost in invasive breast cancer cells [54]. EMT represents a morphological marker of tumor progression, characterized by loss of cell adhesion, repression of E-cadherin expression, and an enhancement of cell mobility and invasiveness. We identified ‘transcriptional regulation by ZEB’ as the third-rank significant pathway and ‘EMT’ as the third-rank significant pathological event relevant to the miR-200b targetome network [17].

### MicroRNA targetome plays a pathological role in Alzheimer’s disease

AD is the most common cause of dementia worldwide, affecting the elderly population, characterized by the hallmark pathology of amyloid-β (Aβ) deposition, neurofibrillary tangle (NFT) formation, and extensive neuronal degeneration in the brain. Aβ is derived from the sequential cleavage of amyloid precursor protein (APP) by beta-site APP-cleaving enzyme 1 (BACE1) and the γ-secretase complex. The hyperphosphorylated tau protein is concentrated in NFT. Although the precise pathological mechanisms underlying AD remain largely unknown, increasing evidence indicates that deregulation of miRNA targetome plays a key role in Aβ production, NFT formation, and neurodegeneration [9,18].

The levels of miR-107 that targets BACE1 are reduced in the temporal cortex not only of AD but also of the patients affected with mild cognitive impairment (MCI), a prodrome of AD, indicating that downregulation of miR-107 begins at the very early stage of AD [55]. The expression of miR-107 is also decreased in the brains of transgenic mice overexpressing human APP carrying familial AD mutations [56]. The expression of miR-29a/b-1 that targets BACE1 is reduced in the anterior temporal cortex of AD, inversely correlated with BACE1 protein levels [57]. A follow-up study showed that the levels of miR-106b that targets APP are also decreased in the anterior temporal cortex of AD [58]. The levels of expression of a noncoding BACE1-antisense (BACE1-AS) RNA that enhances BACE1 mRNA stability are elevated in the brains of Tg19959 APP transgenic mice [59]. Furthermore, BACE1-AS masks the miR-485-5p binding site located within the CDS of BACE1 mRNA, and thereby counteracts miR-485-5p-mediated repression of BACE1 mRNA translation [60]. Actually, the levels of expression of miR-485-5p are reduced but those of BACE1-AS are elevated in the entorhinal cortex and the hippocampus of AD. All of these observations suggest the view that abnormal downregulation of several key miRNAs accelerates β production via overexpression of BACE1, the enzyme and/or APP, the substrate in AD brains [61].

Previously, we found that miR-29a whose levels are decreased in the frontal cortex of AD brains targets neuron navigator 3 (NAV3), a putative axonal guidance regulator [62]. NAV3 immunoreactivity is greatly enhanced in NFT-bearing pyramidal neurons in the cerebral cortex of AD brains, suggesting a compensatory response against NFT-generating neurodegenerative events in neurons. The conditional deletion of Dicer, a master regulator of miRNA processing, induces neurodegeneration accompanied by hyperphosphorylation of tau in the adult mouse forebrain and the hippocampus [63]. Extracellular signal-regulated kinase 1 (ERK1) is identified as a candidate kinase regulated by the miR-15 family responsible for tau phosphorylation. The levels of miR-15a are substantially reduced in AD brains [63].

Recently, we attempted to characterize the miRNA targetome for a battery of miRNAs aberrantly expressed in AD brains [18]. For this purpose, we focused on the currently available most comprehensive dataset of miRNA expression profiling of pathologically validated AD brains [64]. Hierarchical clustering analysis categorized AD-relevant 171 miRNAs into five groups named A to E [64]. We combined them into the set of upregulated miRNAs consisting of groups A and B and the set of downregulated miRNAs consisting of groups C, D, and E. We explored the targets for 171 miRNAs on the miRTarBase [26]. After omitting the mRNAs undetectable in the human brain on UniGene, we extracted 852 theoretically upregulated targets for the set of miRNAs downregulated in AD brains [18].

**Figure 3:**
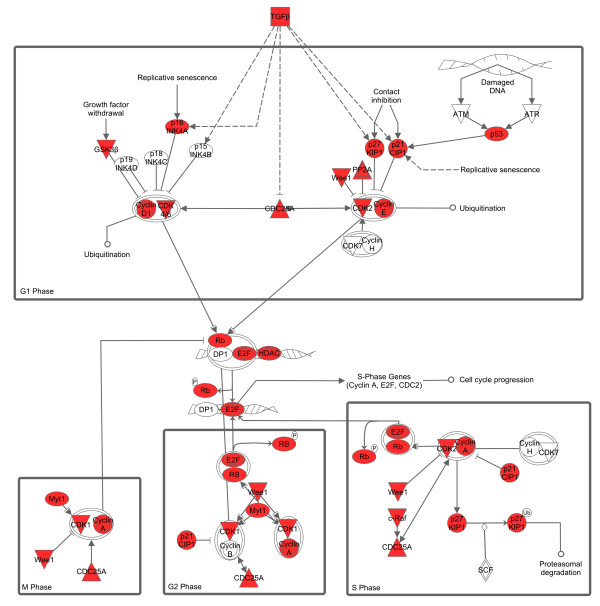
** MicroRNA targetome suggests aberrant upregulation of cell cycle regulators in AD brains.** The targets for the set of miRNAs downregulated in AD brains were identified by searching them on the miRTarBase [18]. The expression of targets in the human brain was verified by searching them on UniGene. Overall, 852 theoretically upregulated target genes for the set of miRNAs downregulated in AD brains were imported into the Core Analysis tool of IPA. The canonical pathway defined by “Cyclins and Cell Cycle Regulation” showing a significant relationship with the targetome (p = 2.18E-19) is shown. The red nodes represent cell cycle regulators theoretically upregulated in AD brains.

Next, we studied molecular networks of 852 targets by using KEGG, IPA, and KeyMolnet. We found a significant association of the targetome with GO terms related to regulation of cell proliferation, cell death, apoptosis, and cell cycle, and KEGG pathways related to cancer, cell cycle, focal adhesion, and signaling pathways of ErbB, p53, MAPK and TGFb [18]. The targetome also showed a significant relationship with IPA functional networks of cancer, cell growth, proliferation, development, and death, and cell cycle, and KeyMolnet pathways of transcriptional regulation by Rb/E2F, cAMP responsive element biding protein (CREB), glucocorticoid receptor (GR), vitamin D receptor (VDR), nuclear factor-kappaB (NF-κB), hypoxia inducible factor (HIF), p53, and AP-1. Collectively, we concluded that the set of miRNAs downregulated in AD brains upregulate the targetome involved in cell cycle progression. The AD-relevant network is characterized by synchronous upregulation of multiple cell cycle regulators, such as cyclins, cyclin-dependent kinases (CDKs), cyclin-dependent kinase inhibitors (CDKIs), Rb/E2F family proteins, and p53 (Figure 3). Our conclusion agrees with previous studies showing that cyclins, CDKs, and CDKIs are aberrantly expressed in AD brains [65,66]. Abnormal reentry into the cell cycle is deleterious for terminally differentiated neurons, and it is identified as an early event in neurons of AD brains, which precedes Aβ deposition and NFT formation [67]. 

## Case study

### in adult T cell leukemia

Finally, we would apply the workflow of molecular network analysis of miRNA targetome to two representative datasets of miRNA expression profiling. As described above, we found that the whole human miRNA targetome network is most closely associated with the disease of adult T cell lymphoma (ATL)/leukemia [17]. Therefore, we focused on a role of miRNA targetome in the pathogenesis of ATL, a highly aggressive T-cell neoplasm caused by human T cell leukemia virus type 1 (HTLV-1). We selected the dataset GSE31629 retrieved from GEO. It contains miRNA expression profiling of peripheral blood mononuclear cells (PBMC) derived form ATL patients (n = 40) and CD4^+^ T cells from healthy control subjects (n = 22). The original study showed that miR-31 that targets NF-κB inducing kinase (NIK) is silenced in ATL cells by an epigenetic mechanism controlled by Polycomb group proteins, leading to persistent activation of NF-κB pathway that plays a central role in ATL leukemogenesis [68]. In their study, PBMCs derived from ATL patients and healthy volunteers were a part of those collected with an informed consent as a collaborative project of the Joint Study on Prognostic Factors of ATL Development (JSPFAD). The project was approved by the Institute of Medical Sciences, the University of Tokyo (IMSUT) Human Genome Research Ethics Committee.

**Figure 4:**
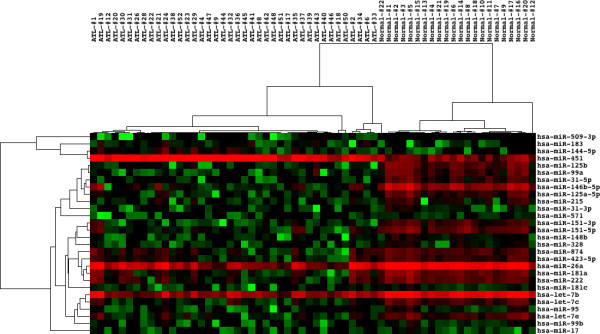
** Differentially expressed microRNAs separate the cluster of ATL cells from normal CD4**^+^**T cells.** We studied the dataset GSE31629 that contains miRNA expression profiling of peripheral blood mononuclear cells (PBMC) derived form ATL patients (n = 40) and CD4^+^ T cells from healthy control subjects (n = 22). Hierarchical clustering analysis of the set of 4 upregulated and 24 downregulated miRNAs in ATL cells versus normal CD4^+^ T cells separated the cluster of ATL samples from the cluster of normal CD4^+^ T cells. Hierarchical clustering analysis was performed by using Cluster 3.0 ( bonsai.ims.u-tokyo.ac.jp/~mdehoon/software/cluster) and TreeView 1.1.5r2 ( sourceforge.net/projects/jtreeview).

First, we extracted the set of 4 upregulated and 24 downregulated miRNAs in ATL cells versus normal CD4^+^ T cells in the condition of p < 0.0001 by Welch’s t-test that corresponds to false discovery rate (FDR) = 0.0085. In ATL cells, upregulated miRNAs include miR-144-5p, 183, 451, and 509-3p, while downregulated miRNAs include let-7b, 7c, 7e, miR-17, 26a, 31-3p, 31-5p, 95, 99a, 99b, 125a-5p, 125b, 146b-5p, 148b, 151-3p, 151-5p, 181a, 181c, 215, 222, 328, 423-5p, 571, and 874. Among the set of 28 miRNAs, 23 miRNAs (82.1%), including miR-31, showed an agreement with the list of deregulated miRNAs reported by the original study [68]. Hierarchical clustering analysis of the set of 28 miRNAs completely separated the cluster of ATL samples from the cluster of normal CD4^+^ T cells (Figure 4). 

**Figure 5:**
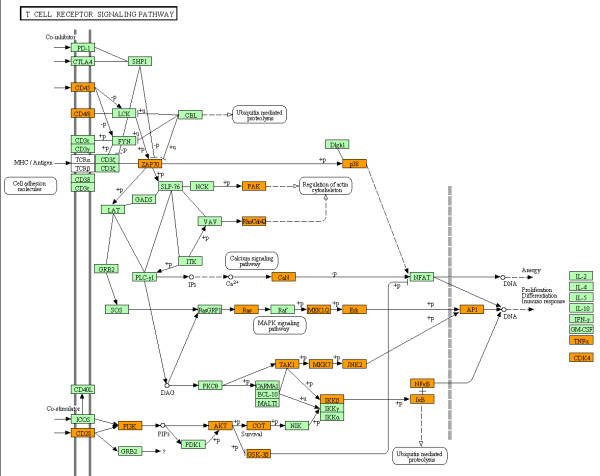
** MicroRNA targetome suggests aberrant upregulation of T cell receptor signaling pathway molecules in ATL cells.** The set of 932 targets for 24 downregluated miRNAs in ATL cells versus normal CD4^+^ T cells (GSE31629) were imported into the Functional Annotation tool of DAVID to identify relevant KEGG pathways. The T-cell receptor (TCR) signaling pathway (hsa04660) (p = 1.31E-5) is shown. The orange nodes represent the genes theoretically upregulated in ATL cells.

Next, we searched validated targets for 28 miRNAs on miRWalk [27] and their expression in leukemia on UniGene. Consequently, we identified 932 targets for downregluated miRNAs and 128 targets for upregulated miRNAs in ATL cells ( Additional file 1: Table S1 and Additional file 2: TableS2). Thus, the targets for downregulated miRNAs greatly outnumbered those of upregulated miRNAs. Thereafter, we focused on the molecular network of 932 targets theoretically upregulated in ATL cells. By importing them into the Functional Annotation tool of DAVID, we found that cancer-related pathways, including the pathway named chronic myeloid leukemia (p = 3.81E-15), were accumulated in the list of top 10 most relevant KEGG pathways. Notably, the T-cell receptor (TCR) signaling pathway was ranked within top 15 (p = 1.31E-5) (Figure 5). 

**Figure 6:**
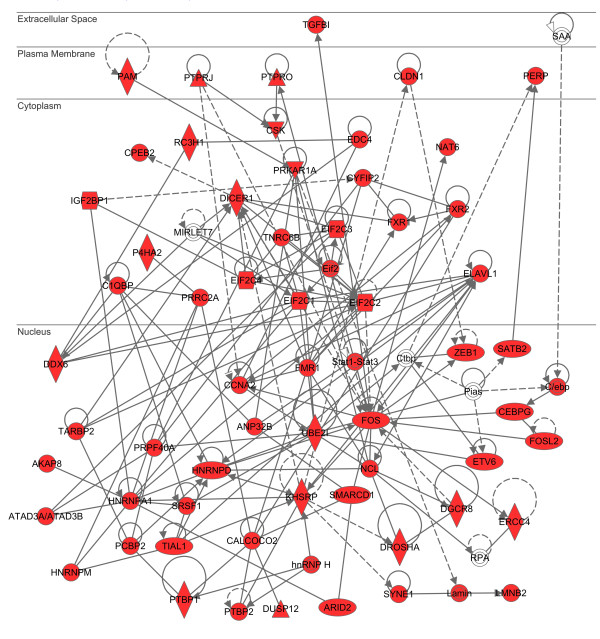
** MicroRNA targetome shows a significant relationship with infection and cancer**-**related network in ATL cells.** The set of 932 targets for 24 downregluated miRNAs in ATL cells versus normal CD4^+^ T cells (GSE31629) were imported into the Core Analysis tool of IPA in the display setting of 70 molecules per network. The first rank molecular network defined by “Infectious Disease, Protein Synthesis, Cancer” (p = 1.00E-82) is shown in the subcellular location layout. The red nodes represent the genes theoretically upregulated in ATL cells.

By using IPA, we found that the network defined by “Infectious Disease, Protein Synthesis, Cancer” (p = 1.00E-82) represented the network most closely related to 932 targets ( Additional file 3: Table S3; Figure 6). By importing 932 genes into KeyMolnet, the neighboring network-search algorithm identified a highly complex network composed of 3,904 molecules and 8,423 molecular relations, showing a significant relationship with canonical pathways of transcriptional regulation by p53 (p = 9.898E-266), Rb/E2F (p = 1.032E-247), CREB (p = 5.084E-203), and SMAD (p = 7.510E-179). Among them, it is well known that CREB acts as a critical regulator of HTLV-1 tax-mediated oncogenesis [69]. These observations suggest that the miRNA targetome aberrantly expressed in ATL cells is shifted to promotion of oncogenesis, possibly triggered by defective TCR signaling attributable to persistent infection of HTLV-1, although this hypothesis awaits experimental validation. 

### Molecular network of MicroRNA targetome in Alzheimer’s disease

By combination of miRNA and mRNA expression profiling of the parietal cortex of AD patients (n = 4) and age-matched controls (n = 4), a recent study showed that the levels of several miRNAs are not only negatively but also positively correlated with those of potential target mRNAs [30]. The expression of miR-211 shows a negative correlation with mRNA levels of BACE1, RAB43, LMNA, MAP2K7, and TADA2L, whereas the expression of mir-44691, a poorly characterized miRNA, exhibits a positive correlation with mRNA levels of CYR61, CASR, POU3F2, GGPR68, DPF3, STK38, and BCL2L2 in AD [30]. We retrieved the dataset of their study numbered GSE16759 from GEO. In their study, postmortem human brain samples were obtained from the University of Southern California (USC) Alzheimer’s Disease Research Center (ADRC), which assures written informed consent from all subjects. The USC Institutional Review Board approved the use of the samples for the study.

**Figure 7:**
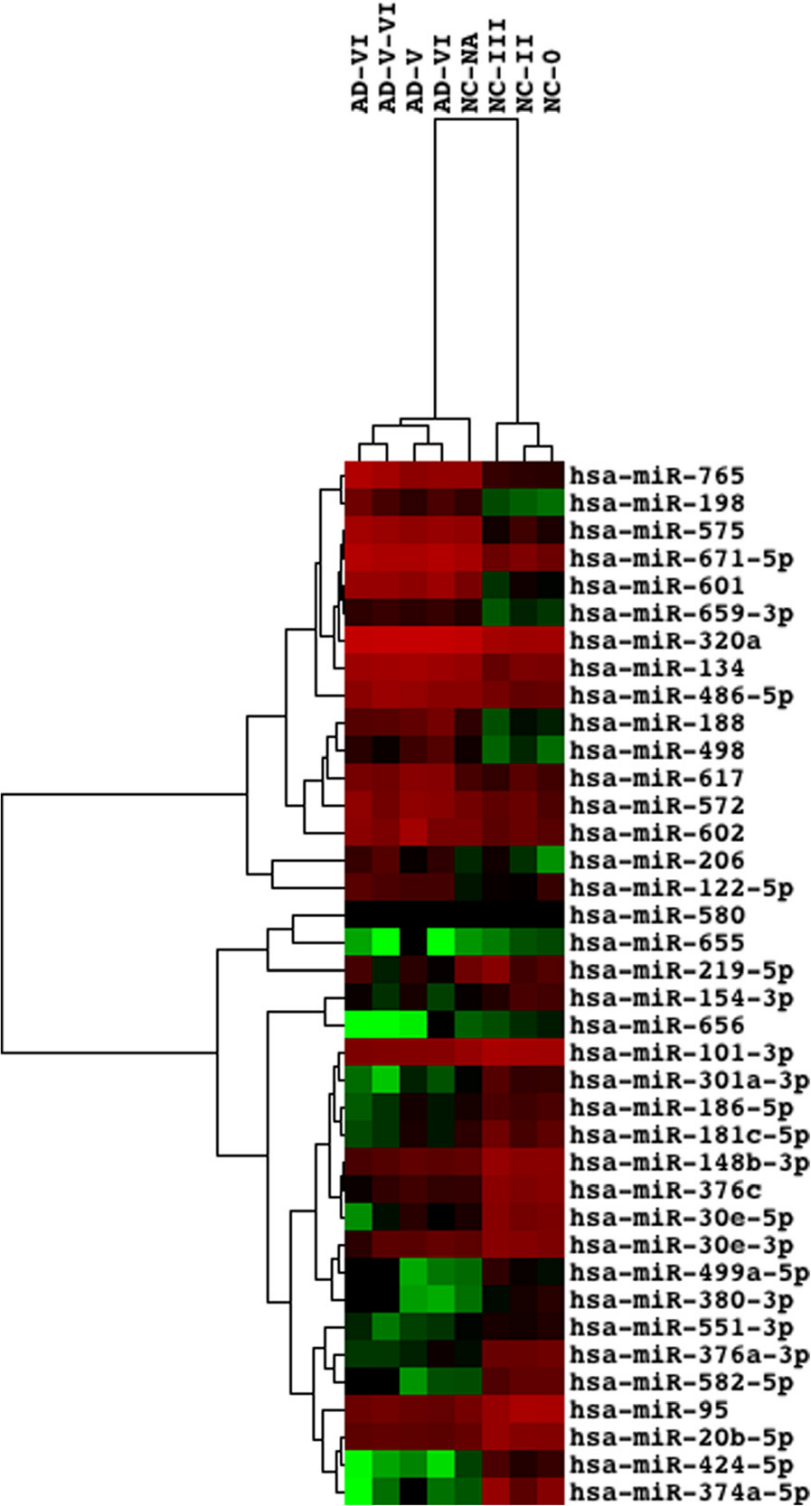
** Differentially expressed microRNAs separate the cluster of AD brains from normal controls.** We studied the dataset GSE16759 that contains miRNA expression profiling of the parietal cortex of AD patients (n = 4) and age-matched normal controls (NC) (n = 4). The Braak staging for AD pathology ranking from 0 to VI is shown in individual cases. Hierarchical clustering analysis of the set of 16 upregulated and 22 downregulated miRNAs in AD versus NC separated the cluster of AD samples from the cluster of NC, except for one NC sample classified as an intermediate between AD and NC groups. Hierarchical clustering analysis was performed by using Cluster 3.0 and TreeView 1.1.5r2.

First, we extracted the set of 16 upregulated and 22 downregulated miRNAs in AD brains versus normal brains in the condition of p < 0.05 by Welch’s t-test and fold change greater than 3. In AD brains, upregulated miRNAs include miR-122-5p, 134, 188, 198, 206, 320a, 486-5p, 498, 572, 575, 601, 602, 617, 659-3p, 671-5p, and 765, while downregulated miRNAs include miR-20b-5p, 30e-3p, 30e-5p, 95, 101-3p, 148b-3p, 154-3p, 181c-5p, 186-5p, 219-5p, 301a-3p, 374a-5p, 376a-3p, 376c, 380-3p, 424-5p, 499a-5p, 551-3p, 580, 582-5p, 655, and 656. Hierarchical clustering analysis of the set of 38 miRNAs separated the cluster of AD samples from the cluster of normal control (NC) subjects, except for one NC sample classified as an intermediate between AD and NC groups (Figure 7).

**Figure 8:**
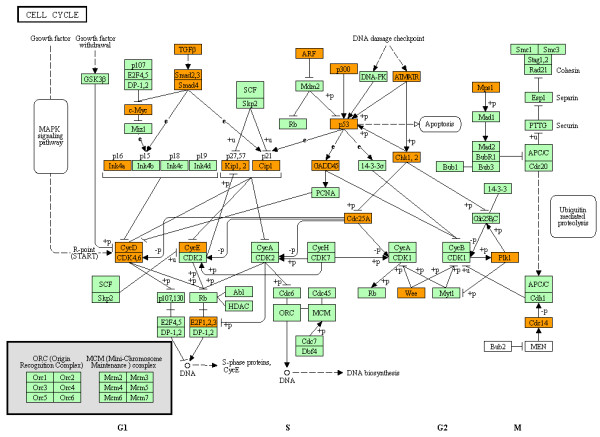
** MicroRNA targetome suggests aberrant upregulation of cell cycle regulators in AD brains.** The set of 662 targets for 22 downregluated miRNAs in AD brains versus normal control brains (GSE16759) were imported into the Functional Annotation tool of DAVID to identify relevant KEGG pathways. The cell cycle pathway (hsa04110) (p = 1.51E-5) is shown. The orange nodes represent the genes theoretically upregulated in AD brains.

Next, we searched validated targets for 38 miRNAs on miRWalk and their expression in brain on UniGene. Overall, we identified 662 targets for downregluated miRNAs and 265 targets for upregulated miRNAs in AD brains ( Additional file 4: Table S4 and Additional file 5: Table S5). The analysis of mRNA expression data corresponding to miRNA expression profiling identified 53 differentially expressed mRNAs. However, only three transcripts, such as Ras homolog enriched in brain (RHEB) with a 0.47-fold change, chemokine (C-X-C motif) ligand 12 (CXCL12) with a 2.06-fold change, and podocalyxin-like (PODXL) with a 2.05-fold change, showed an agreement with those in the list of miRNA targets, being consistent with the view that the miRNA-target mRNA interaction often causes translational repression without changing mRNA levels. Thereafter, we focused on the molecular network of 662 targets theoretically upregulated in AD brains. By importing them into the Functional Annotation tool of DAVID, we found that several cancer-related pathways, MAPK signaling pathway, and cell cycle pathway (p = 1.51E-5) (Figure 8) were accumulated in top 10 most relevant KEGG pathways (Table 1).

**Table 1:** Top 10 KEGG pathways associated with 662 miRNA targets theoretically upregulated in AD brains

**Rank**	**Category**	**Genes**	**Bonferroni**-**Corrected p**-**Value**	**FDR**
1	hsa05200:Pathways in cancer	ACVR1B, ACVR1C, AKT1, APC, BCL2, BCR, CASP3, CCND1, CCNE1, CCNE2, CDK6, CDKN1A, CDKN1B, CDKN2A, CEBPA, CTNNB1, CUL2, E2F1, E2F3, EGFR, EP300, EPAS1, ERBB2, ETS1, FGF13, FGF2, FGFR1, FH, FLT3, FOS, FOXO1, HGF, HIF1A, HRAS, IL6, ITGA2, ITGA2B, JUN, KRAS, LAMC1, MAP2K1, MAPK8, MAPK9, MET, MMP9, MTOR, MYC, PDGFA, PIAS1, PIK3CA, PML, PPARG, PRKCA, PTEN, PTGS2, RARA, RASSF1, RUNX1, RXRA, SHH, SMAD2, SMAD3, SMAD4, STAT1, STAT3, TGFB1, TGFBR1, TGFBR2, TP53, TPM3, VEGFA, WNT5A, XIAP	2.84E-21	2.20E-20
2	hsa05212:Pancreatic cancer	ACVR1B, ACVR1C, AKT1, CCND1, CDK6, CDKN2A, E2F1, E2F3, EGFR, ERBB2, KRAS, MAP2K1, MAPK8, MAPK9, PIK3CA, SMAD2, SMAD3, SMAD4, STAT1, STAT3, TGFB1, TGFBR1, TGFBR2, TP53, VEGFA	3.06E-10	2.37E-09
3	hsa05210:Colorectal cancer	ACVR1B, ACVR1C, AKT1, APC, BCL2, CASP3, CCND1, CTNNB1, EGFR, FOS, JUN, KRAS, MAP2K1, MAPK8, MAPK9, MET, MYC, PIK3CA, SMAD2, SMAD3, SMAD4, TGFB1, TGFBR1, TGFBR2, TP53	1.25E-08	9.70E-08
4	hsa05220:Chronic myeloid leukemia	ACVR1B, ACVR1C, AKT1, BCR, CCND1, CDK6, CDKN1A, CDKN1B, CDKN2A, E2F1, E2F3, HRAS, KRAS, MAP2K1, MYC, PIK3CA, RUNX1, SMAD3, SMAD4, TGFB1, TGFBR1, TGFBR2, TP53	4.51E-08	3.50E-07
5	hsa05215:Prostate cancer	AKT1, BCL2, CCND1, CCNE1, CCNE2, CDKN1A, CDKN1B, CREB1, CTNNB1, E2F1, E2F3, EGFR, EP300, ERBB2, FGFR1, FOCO1, HRAS, KRAS, MAP2K1, MTOR, PDGFA, PIK3CA, PTEN, TP53	2.83E-07	2.19E-06
6	hsa05219:Bladder cancer	CCND1, CDKN1A, CDKN2A, E2F1, E2F3, EGFR, ERBB2, HRAS, KRAS, MAP2K1, MMP9, MYC, RASSF1, THBS1, TP53, VEGFA	1.66E-06	1.29E-05
7	hsa05218:Melanoma	AKT1, CCND1, CDK6, CDKN1A, CDKN2A, E2F1, E2F3, EGFR, FGF13, FGF2, FGFR1, HGF, HRAS, KRAS, MAP2K1, MET, PDGFA, PIK3CA, PTEN, TP53	4.12E-06	3.19E-05
8	hsa04010:MAPK signaling pathway	ACVR1B, ACVR1C, AKT1, ATF2, BDNF, CACNA1C, CACNA2D1, CACNB1, CASP3, DUSP1, DUSP16, EGFR, FGF13, FGF2, FGFR1, FOS, GADD45A, HRAS, HSPA1A, HSPA1B, JUN, KRAS, MAP2K1, MAP3K12, MAPK14, MAPK8, MAPK9, MAPKSP1, MEF2C, MYC, NLK, PDGFA, PPP3CA, PPP3R1, PPP5C, PRKCA, RASA1, STMN1, TGFB1, TGFBR1, TGFBR2, TP53	1.17E-05	9.03E-05
9	hsa04110:Cell cycle	ATM, CCND1, CCND2, CCND3, CCNE1, CCNE2, CDC14A, CDC25A, CDK6, CDKN1A, CDKN1B, CDKN2A, CHEK1, E2F1, E2F3, EP300, GADD45A, MYC, PLK1, SMAD2, SMAD3, SMAD4, TGFB1, TP53, TTK, WEE1	1.51E-05	1.17E-04
10	hsa05214:Glioma	AKT1, CAMK2G, CCND1, CDK6, CDKN1A, CDKN2A, E2F1, E2F3, EGFR, HRAS, KRAS, MAP2K1, MTOR, PDGFA, PIK3CA, PRKCA, PTEN, TP53	1.99E-05	1.54E-04

By importing Entrez Gene IDs of 662 miRNA target genes theoretically upregulated in AD brains (GSE16759) into the Functional Annotation tool of DAVID, top 10 most relevant KEGG pathways were identified. They are listed with Category, Genes, Bonferroni-corrected p-value of the modified Fisher’s exact test, and false discovery rate (FDR).

By using IPA, we identified molecular networks with functional categories defined by “Cancer, Reproductive System Disease, Cell Cycle” (p = 1.00E-76) and “Gene Expression, Cell Cycle, DNA Replication, Recombination, and Repair” (p = 1.00E-72) as the networks most closely related to 662 targets. By importing 662 genes into KeyMolnet, the neighboring network-search algorithm identified a highly complex network composed of 3,255 molecules and 6,133 molecular relations, showing a significant relationship with canonical pathways of transcriptional regulation by p53 (p = 1.136E-283), SMAD (p = 1.167E-252), CREB (p = 6.075E-222), and Rb/E2F (p = 2.173E-199), all of which play a pivotal role in cell cycle regulation. These observations suggest that the miRNA targetome aberrantly expressed in AD brains is shifted to deregulation of cell cycle that plays a central role in the pathogenesis of AD, being consistent with our recent observations [18]. Therefore, this hypothesis warrants experimental validation.

## Concluding remarks

A single miRNA concurrently downregulates hundreds of target mRNAs [2,4]. Such fuzzy miRNA-mRNA interactions contribute to the complexity and the redundancy of miRNA-regulated targets and their networks. Recently, we found that the miRNA targetome regulated by an individual miRNA generally constitutes the biological network of functionally-associated molecules in human cells, closely linked to pathological events involved in cancers and neurodegenerative diseases [17,18]. Increasing evidence supports this view. Interacting proteins in the human PPI network often share restricted miRNA target-site types than random pairs [70]. A computational method named mirBridge, which assesses enrichment of functional sites for a given miRNA in the annotated gene set, showed that various miRNAs coordinately regulate multiple components of signaling pathways and protein complexes [71].

We identified a coordinated regulation of gene expression by transcription factors and miRNAs at transcriptional and posttranscriptional levels in cancer-associated miRNA targetome networks [17]. Positive and negative transcriptional coregulation of miRNAs and their targets plays a crucial role in conferring robustness to the gene regulatory networks in mammalian genomes [14,72]. For example, the protooncogene c-myc directly activates transcription of E2F1, but at the same time limits its translation by upregulating expression of miR-17-5p and miR-20a, both of which negatively regulate E2F1 [73]. Importantly, the genes with more transcription factor-binding sites have a higher probability of being targeted by miRNAs and have more miRNA-binding sites [74].

We found that the most relevant pathological event in the whole human miRNA targetome is ‘cancer’, supporting the general view that the human miRNAome plays a specialized role in regulation of oncogenesis [17]. Many miRNA gene loci are clustered in cancer-associated genomic regions [75]. Furthermore, miRNA expression signatures clearly discriminate different types of cancers with distinct clinical prognoses [76]. By miRNA expression profiling of thousands of human tissue samples, a recent study showed that diverse sets of miRNAs constitute a complex network composed of coordinately regulated miRNA subnetworks in both normal and cancer tissues, and they are often disorganized in solid tumors and leukemias [77]. During oncogenesis, various panels of miRNAs act as either oncogenes named oncomir or tumor suppressors termed anti-oncomir, or both, by targeting key molecules and their networks involved in apoptosis, cell cycle, cell adhesion and migration, chromosome stability, and DNA repair [8].

In the present review, we applied the workflow of molecular network analysis of miRNA targetome to two representative datasets of miRNA expression profiling, such as ATL and AD. The results supported the view that miRNAs act as a central regulator of both oncogenesis and neurodegeneration. Therefore, the miRNA-based therapy designed to target simultaneously multiple cancer-associated or neurodegenerative networks and pathways might provide a rational and effective approach to treating and preventing cancers and AD.

## Competing interests

The author declares no competing interests.

## Authors’ contributions

JS performed all data analyses and drafted the manuscript.

## Supplementary Material

Additional file 1 ** Table S1.** The list of 128 target mRNAs for upregulated miRNAs in ATL cells. Click here for file

Additional file 2 ** Table S2.** The list of 932 target mRNAs for downregulated miRNAs in ATL cells. Click here for file

Additional file 3 ** Table S3.** The list of top 10 IPA molecular networks relevant to 931 miRNA targets theoretically upregulated in ATL cells. Click here for file

Additional file 4 ** Table S4.** The list of 265 target mRNAs for upregulated miRNAs in AD brains. Click here for file

Additional file 5 ** Table S5.** The list of 662 target mRNAs for downregulated miRNAs in AD brains. Click here for file
